# Supplementary material for: Occurrence of No-Harm Incidents and Adverse Events in Hospitalized Patients with Ischemic Stroke or TIA: A Cohort Study Using Trigger Tool Methodology
Source: Int J Environ Res Public Health. 2022 Feb 27;19(5):2796. doi: 10.3390/ijerph19052796 (PMC8910044; doi:10.3390/ijerph19052796)
Supplement: Supplementary file 1 [file ijerph-19-02796-s001.zip › ijerph-1571923-supplementary.pdf]

Supplementary Materials S1

Education

The two primary reviewers, B.N. and L.S. (both registered nurses), conducted the Institute for Healthcare Improvement's (IHI's) Global Trigger Tool (GTT) education module and studied the related whitepaper [36]. A pre-test with 20 electronic health records was performed for training purposes and to optimize the study-specific screening protocols, which were developed via an iterative process. B.N. briefed the four participating senior stroke physicians regarding the GTT methodology. The review process was supervised by an external GTT expert (M.U.) to support adherence to the methodology.

Interrater reliability

Consensus between primary reviewers was determined on the level of event agreement to determine inter-rater reliability via weighted Cohen`s Kappa. Inter-rater reliability between the two primary reviewers on the level of the total number of identified events per admission showed a weighted Cohen`s Kappa of 0.61, indicating good reviewer agreement.

Screening time

The time taken for primary review differed between the two reviewers. The first reviewer spent a total of 19 hours and 15 minutes screening all 150 electronic health records, with a median time of 13 minutes per chart (IQR: 8 minutes and 31 seconds – 21 minutes and 31 seconds). The second reviewer spent a total screening time of 9 hours and 42 minutes, with a median of 10 minutes per chart (IQR: 6 minutes and 2 seconds – 16 minutes and 56 seconds). The consensus process took 20 hours and 24 minutes in total, with a median of 5 minutes per file (range: 10 seconds – 20 minutes). Due to our intention to identify any possible event and not collecting the AEs as part of routine quality improvement work, we didn't apply the 20-minutes screening time [12].

Trigger occurrence and positive predictive value

Overall, 509 triggers were found in the 150 electronic health records investigated, with a mean of 3.4 triggers (range 0 – 21) per record. No triggers were found in 21 records. The total positive predictive value (PPV) for adverse events (AEs) and no-harm incidents was 39.7% including 202 triggers. The trigger *other* was used 150 (29.5%) times during primary review, making it the most frequent trigger with a total PPV of 32.0%.

The total PPV for AE-related triggers was 27.3% (range: 0% – 100%). The triggers most frequently related to AEs, and which showed the highest PPV were *in-hospital stroke* (n=11, PPV 100%), *healthcare-associated infections* (n=15, PPV 100%), *any procedure complications* (n=36, PPV 81.8%). The PPVs for triggers related to no-harm incident ranged from 0% - 100%, with a total of 12.4%. With an occurrence of 37 triggers the trigger *other* from the medication module showed the highest PPV of 100%. A detailed overview of trigger occurrence, along with their associated PPVs and total PPVs, is provided in the table S1.

There is a difference between the number of patients, who received an endovascular treatment during acute treatment (n=10) and the number of triggers being used for endovascular treatment during primary review (n=20). The difference is explained by some patients receiving endovascular treatment for diagnostic or therapeutic purposes during their inpatient stay. There is also a difference between the

number of patients, who received a systematic administration of rtPA during acute treatment (n=26) and the number of triggers being used for rtPA administration (n=27). One patient received acute treatment with rtPA in another hospital before the transfer to our hospital some days later. Since this patient did not receive the acute treatment at our hospital, it does not appear in the demographic patient data, but it was recorded in the triggers.

Table S1. Frequency of trigger occurrence and positive predictive value, sorted by the column PPV total

	No [1] of triggers detected in primary review	No of triggers related to AE [3]	PPV [2] for triggers related to AE, %	No of triggers related to NHI [4]	PPV of triggers related to NHI, %	PPV total, %
**Care module**						
In-hospital stroke	11	11	100	0	0	100
Healthcare-associated infections	15	15	100	0	0	100
Pressure ulcers	4	4	100	0	0	100
Any procedure complication	44	36	81.8	6	13.6	95.5
Transfusion of blood or use of blood products	4	2	50.0	0	0	50.0
Other	150	36	24.0	12	8.0	32.0
Patient fall	20	2	10.0	6	30.0	40.0
Restraint use	5	2	40.0	0	0	40.0
Decrease in hemoglobin or hematocrit of 25% or greater	6	1	16.7	0	0	16.7
Readmission within 30 days	45	7	15.6	0	0	15.6
Transfer to higher level of care	25	2	8.0	0	0	8.0
Positive blood culture	0	0	0	0	0	0
Cardiac or pulmonary arrest, or rapid response team activation	0	0	0	0	0	0
Acute dialysis	0	0	0	0	0	0
X-Ray or doppler studies for emboli or deep vein thrombosis	2	0	0	0	0	0
**Medication module**						
Other	37	0	0	37	100	100
Over-sedation/hypotension	3	3	100	0	0	100
Abrupt medication stop	5	1	20.0	1	20.0	40.0
Rising blood urea nitrogen or serum creatinine two times (2x) over baseline	3	1	33.3	0	0	33.3
Diphenhydramine (Benadryl) administration	5	1	20	0	0	20.0
Anti-emetic administration	12	1	8.3	0	0	8.3
Clostridium difficile positive stool	0	0	0	0	0	0
Partial thromboplastin time greater than 100 seconds	3	0	0	0	0	0
International normalized ratio (INR) greater than 6	1	0	0	0	0	0
Glucose less than 50mg [5]/dl [6]	0	0	0	0	0	0
Vitamin K administration	3	0	0	0	0	0
Romazicon (Flumazenil) administration	0	0	0	0	0	0
Naloxone (Narcan) administration	0	0	0	0	0	0
**(self-developed) Stroke module**						
EVT [7]	20	6	30.0	1	5.0	35.0
Neurological decline of the GCS [8] $\geq$ 4 from the intimal score	4	1	25.0	0	0	25.0
Systemic administration of rtPA [9]	27	5	18.5	0	0	18.5
Neurological decline of the NIHSS [10] $\geq$ 4 from the initial score	19	2	10.5	0	0	10.5
Thrombin time 1 $\leq$ 120 seconds and thrombin time 2 $\leq$ 4-8 seconds while under therapeutic heparin within 24 hours from onset	32	0	0	0	0	0
Systolic blood pressure above 185mmHg [11] during rtPA administration or in	3	0	0	0	0	0

accordance with the neuroradiological report						
Computer tomography brain scan ≤ 12 hours after rtPA administration	1	0	0	0	0	0
Administration of coagulation factors	0	0	0	0	0	0
**Total**	**509**	**139**	**27.3**	**63**	**12.4**	**39.7**

Abbreviations: [1] No number, [2] AE adverse event, [3] NHI no-harm incident, [4] PPV positive predictive value, [5] mg milligram, [6] dl deciliter, [7] EVT endovascular treatment, [8] GCS Glasgow Coma Scale, [9] rtPA recombinant tissue plasminogen activator, [10] NIHSS National Institution of Health Stroke Scale, [11] mmHg millimeter of mercury

There are several explanations for the high use of the trigger *other* in the care module. One reason is, that we additionally collected no-harm incidents, even though they are not explicit recommended by the GTT manual such as non-performed tasks, falls without injury etc. Another reason is, that the care module of the GTT doesn't provide triggers, which explicitly refer to a specific event type. Many of the identified events e.g., delirium, severe constipation, severe diarrhea, severe not controlled pain, local hematoma, rash were detected by using the trigger *other*, because no other suitable trigger exists in the origin Global Trigger Tool.

Common events not explicitly represented by a specific trigger in the care module may led to missing events during record review, such as e.g., urinary retention or infiltrated intravenous infusion [37]. Therefore, it is common, to add explicit triggers, e.g., blood vessel, skin and/or tissue harm, moderate/severe agitation and/or acute confusion/delirium, distended urinary bladder, neurological impairment and/or harm to make sure not to miss common events when the trigger *other* is the only option [12,22,24]. This may indicate the need for a further development of the GTT`s care module with more explicit triggers for common event types since the development of new triggers and trigger tools have increased rapidly since the last revision of the GTT manual over a decade ago.

Detailed event presentation

All events, which were found during the review process, are listed in table S2. To represent a broad patient perspective, we collected any events and assigned them based on their origin to stroke services related events or other care services related events. Stroke service-related events included any that took place during the initial stroke admission during the whole stroke care. For example, if a stroke patient fell off a stretcher while waiting for a stroke-specific diagnostic procedure, this was rated as a stroke service-related event. On the other hand, any event that was detected during the initial stroke admission, but was allocated outside the stroke treatment, e.g., if a patient (not admitted for stroke) suffered a stroke during coronary angiography this was rated as an AE related to other care services.

Table S2. Detailed event presentation; sorted by column total

	**Stroke service-related (N=142)**		**Other health service-related (N=28)**		**Total (N=170)**
	**NHI [1]**	**AEs [2]**	**NHI**	**AEs**	**NHIs and AEs**
**General care-related events, n**	**15**	**23**	**2**	**6**	**46**
**(%; 95% CI)**	**(26.3; 15.9-39.9)**	**(27.1; 18.3-38.0)**	**(40.0; 7.2-83.0)**	**(26.1; 11.1-48.7)**	**(27.1; 20.7-34.5)**
Phlebitis, n (%)		9 (39.1)		1 (16.7)	10 (21.7)
Others, n (%)	4 (26.7)	2 (8.7)	1 (50.0)	1 (16.7)	8 (17.4)
Severe constipation, n (%)		4 (17.4)		3 (50)	7 (15.2)
Non-performed task*, n (%)	6 (40)		1 (50.0)		7 (15.2)
Severe, not controlled pain, n (%)		2 (8.7)		1 (16.7)	3 (6.5)
Moderate diarrhea, n (%)	2 (13.3)				2 (4.3)
Severe diarrhea, n (%)		2 (8.7)			2 (4.3)
Moderate constipation, n (%)	1 (6.7)				1 (2.2)
Urinary retention, n (%)	1 (6.7)				1 (2.2)
Non-removed catheters, n (%)	1 (6.7)				1 (2.2)
Eczema, n (%)		1 (4.3)			1 (2.2)
Intertrigo, n (%)		1 (4.3)			1 (2.2)
Dehydration, n (%)		1 (4.3)			1 (2.2)
Over-sedation, n (%)		1 (4.3)			1 (2.2)
**Medication events, n**	**35**	**-**	**2**	**-**	**37**
**(%; 95% CI)**	**(61.4; 47.6-73.7)**		**(40.0, 7.2-83.0)**		**(21.8; 16.0-28.9)**
Wrong prescription, n (%)	16 (45.7)		1 (50.0)		17 (45.9)
Other medication event, n (%)	4 (11.2)		1 (50.0)		5 (13.5)
Wrong administration, n (%)	4 (11.4)				4 (10.8)
Not prescribed, n (%)	4 (11.2)				4 (10.8)
Omission, n (%)	3 (8.6)				3 (8.1)

Prescription delay, n (%)	2 (5.7)				2 (5.4)
Wrong preparation, n (%)	1 (2.9)				1 (2.7)
Wrong documentation, n (%)	1 (2.9)				1 (2.7)
**Neurologic events, n**	**1**	**22**	**-**	**7**	**30**
**(%; 95% CI)**	**(1.8; 0.1-10.6)**	**(25.9; 17.3-36.7)**		**(30.4; 14.1-53.0)**	**(17.6; 12.4-24.4)**
Stroke, n (%)		7 (31.8)		5 (71.4)	12 (40)
(new) Stroke, n (%)		4 (18.2)		5 (71.4)	9 (30)
(re) Stroke, n (%)		3 (13.6)			3 (10)
Delirium, n (%)		6 (27.3)			6 (20)
Other cerebrovascular events, n (%)		4 (18.2)		1 (14.3)	5 (16.7)
Intracerebral parenchymal bleeding, n (%)		3 (13.6)			3 (10)
TIA [3], n (%)				1 (14.3)	1 (3.3)
Subdural bleeding, n (%)		1 (4.5)			1 (3.3)
Epilepsia, n (%)		1 (4.5)			1 (3.3)
Brain artery perforation without SAB [4], n (%)	1 (100)				1 (3.3)
**Healthcare-associated infections, n**	**-**	**11**	**-**	**4**	**15**
**(%; 95% CI)**		**(12.9; 6.9-22.4)**		**(17.4; 5.7-39.5)**	**(8.8; 5.2-14.4)**
Urinary tract infection, n (%)		9 (81.8)		2 (50)	11 (73.3)
Upper respiratory tract infection, n (%)		1 (9.1)		1 (25)	2 (13.3)
Erysipelas, n (%)		1 (9.1)			1 (6.7)
Infection; focus unknown, n (%)				1 (25)	1 (6.7)
**Bleedings, n**	**-**	**9**	**-**	**2**	**11**
**(%; 95% CI)**		**(10.6; 5.2-19.6)**		**(8.7; 1.5-29.5)**	**(6.5; 3.4-11.6)**
Epistaxis, n (%)		3 (33.3)			3 (27.3)
Gastrointestinal bleeding, n (%)		2 (22.2)			2 (18.2)
Macrohaematuria, n (%)		2 (22.2)			2 (18.2)
Gingival, n (%)		1 (11.1)		1 (50)	2 (18.2)
Local hematoma, n (%)		1 (11.1)			1 (9.1)
Other types of bleeding, n (%)				1 (50)	1 (9.1)
**Internal events, n**	**1**	**9**	**-**	**1**	**11**
**(%; 95% CI)**	**(1.8; 0.1-10.6)**	**(10.6; 5.3-19.6)**		**(4.3; 0.2-24.0)**	**(6.5; 3.4-11.6)**
Statin associated myalgia/myopathy, n (%)		3 (33.3)			3 (37.3)
Symptomatic hypotension, n (%)		2 (22.2)			2 (18.2)
Renal insufficiency, n (%)		2 (22.2)			2 (18.2)
Cardiac decompensation, n (%)		1 (11.1)		1 (100)	2 (18.2)
Hepatopathy, n (%)		1 (11.1)			1 (9.1)

Pre-syncope, n (%)	1 (100)				1 (9.1)
**Allergic reactions, n**	**-**	**7**	**-**	**1**	**8**
**(%; 95% CI)**		**(8.2; 3.7-16.8)**		**(4.3; 0.2-24.0)**	**(4.7; 2.2-9.4)**
Rash, n (%)		5 (71.4)			5 (62.5)
Anaphylactic reaction, n (%)		1 (14.3)			1 (12.5)
Other allergic reactions, n (%)		1 (14.3)		1 (100)	2 (25)
**Falls, n**	**5**	**1**	**1**	**1**	**8**
**(%; 95% CI)**	**(8.8; 3.3-20.0)**	**(1.2; 0.1-7.3)**	**(20.0; 1.1-70.1)**	**(4.3; 0.2-24.0)**	**(4.7; 2.2-9.4)**
Without injury, n (%)	5 (100)		1 (100)		6 (75)
Local contusion/hemorrhage, n (%)		1 (100)		1 (100)	2 (25)
**Pressure ulcers, category I, n**	**-**	**3**	**-**	**1**	**4**
**(%; 95% CI)**		**(3.5; 0.9-10.7)**		**(4.3; 0.2-24.0)**	**(2.4; 0.8-6.3)**
**Total, n (%)**	**57 (33.5)**	**85 (50.0)**	**5 (2.9)**	**23 (13.5)**	**170 (100)**

Abbreviations: [1] NHI non-harm incident, [2] AE adverse event, [3] TIA transitory ischemic attack, [4] SAB subarachnoid bleeding,

*Non-performed tasks include monitoring/diagnostics/laboratory/preoperative preparation
